# Rare central nervous system lymphomas

**DOI:** 10.1111/bjh.18128

**Published:** 2022-03-16

**Authors:** Furqaan Ahmed Kaji, Nicolás Martinez‐Calle, Vishakha Sovani, Christopher Paul Fox

**Affiliations:** ^1^ Clinical Haematology Nottingham University Hospitals NHS Trust Nottingham UK; ^2^ Department of Histopathology Nottingham University Hospitals NHS Trust Nottingham UK

**Keywords:** CNS, Hodgkin lymphoma, lymphomas, non‐Hodgkin lymphoma

## Abstract

Central nervous system (CNS) lymphomas are rare malignancies characterised by lymphoid infiltration into the brain, spinal cord, cranial nerves, meninges and/or eyes in the presence or absence of previous or concurrent systemic disease. Most CNS lymphomas are of the diffuse large B‐cell lymphoma (DLBCL) subtype for which treatment strategies, particularly the use of high‐dose methotrexate‐based protocols and consolidation with autologous stem cell transplantation, are well established. Other histopathological subtypes of CNS lymphoma are comparatively less common with published data on these rare lymphomas dominated by smaller case series and retrospective reports. Consequently, there exists little clinical consensus on the optimal methods to diagnose and manage these clinically and biologically heterogeneous CNS lymphomas. In this review article, we focus on rarer CNS lymphomas, summarising the available clinical data on incidence, context, diagnostic features, reported management strategies, and clinical outcomes.

## INTRODUCTION

Central nervous system (CNS) lymphomas are rare haematological malignancies. Primary CNS lymphomas (PCNSL) account for ~4% of all brain tumours and are formally classified by the World Health Organisation as diffuse large B‐cell lymphoma (DLBCL) isolated to the CNS (brain, spinal cord, cranial nerves, meninges, and/or eyes) without systemic involvement.^1^, ^2^ Secondary CNS lymphomas (SCNSL) are also typically DLBCL. These can present as synchronous systemic and CNS disease (at initial diagnosis or at recurrence) or as an isolated CNS relapse following previous treatment for systemic DLBCL.

The biological mechanisms underlying the tropism of DLBCL for the CNS are not fully understood.^3^, ^4^ Despite this, management strategies for primary and secondary CNS‐DLBCLs are relatively well characterised within the literature. Current consensus involves treating primary CNS‐DLBCL with induction chemoimmunotherapy incorporating a high‐dose methotrexate (HD‐MTX) backbone.^5^, ^6^ For suitably fit patients, remission induction treatment is followed by consolidation with high‐dose thiotepa‐based autologous stem cell transplantation (HDT‐ASCT) or whole brain radiotherapy (WBRT).^6^, ^7^ More recently, the MARIETTA study (ClinicalTrials.gov Identifier: NCT02329080) for patients with secondary CNS‐DLBCL described similarly intensive CNS‐directed chemotherapy and consolidation ASCT as an effective approach for this group of patients.^8^

By comparison to DLBCL, other histopathological subtypes of CNS lymphoma are rare, with the published literature dominated by observational studies and isolated case reports describing these pathological entities. There is a lack of consensus on how to approach the diagnosis and management of this heterogeneous group of rare CNS lymphomas.

We undertook a literature review and narrative synthesis of the published data on rare CNS lymphomas, with the intention to provide a concise summary of the incidence, diagnostic features and challenges, management options, and anticipated clinical outcomes before suggesting a pragmatic clinical approach to these lymphoma entities. In order to address uncertainties presented by inconsistent staging procedures and to encompass a breadth of clinical scenarios, we included both rare primary CNS lymphomas and rare secondary CNS lymphomas, where the CNS presentation is described as the dominant clinical problem. Importantly, for many of these rare lymphoma subtypes, there is a paucity of high‐quality evidence informing clinical management strategies and we recognise the risk of positive reporting bias. As such, definitive conclusions on optimal treatment approaches are not possible and, hence, interpretation of published data also reflects consensus and clinical experience of the authors of this article.

## DIAGNOSTIC APPROACHES AND CHALLENGES FOR CNS LYMPHOMAS

Cytological evaluation of cerebrospinal fluid (CSF) is a relatively straightforward diagnostic procedure but provides low sensitivity for diagnosis and typically does not permit accurate subclassification of CNS lymphomas. The combined sensitivity of CSF cytology, flow cytometry, CSF lactate dehydrogenase (LDH) isozyme 5, β2‐microglobulin, and immunoglobulin heavy (IGH) chain rearrangement studies (for B‐cell lymphomas) is superior to CSF cytology alone but even this integrated approach provides only moderate specificity.^9^ More recently, there has been increased interest in the use of circulating tumour DNA (ctDNA) to aid diagnosis across lymphoma subtypes and improve the sensitivity of detecting disease recurrence.^10^, ^11^ A specific example is the diagnostic utility of the myeloid differentiation primary response 88 (*MYD88*) L265P mutation given its presence in >80% of PCNSL cases. Indeed, one small study detected this mutation in the CSF ctDNA of 20/26 patients with CNS lymphoma.^12^ Importantly, ctDNA analysis does not distinguish between DLBCL and lymphoplasmacytic histological subtypes.^11^ Nevertheless, ctDNA (from blood and/or CSF) holds much promise for the diagnosis of CNS lymphoma and is likely to provide additional contributions to the differential diagnoses of less common CNS lymphoma entities.

The current diagnostic ‘gold standard’ remains histopathological diagnosis following biopsy of a CNS lesion. Nonetheless, biopsy poses a number of challenges, namely the procedural risks together with the diagnostic challenges presented by typically tiny fragments of tumour tissue. It is also well recognised that administration of corticosteroids prior to biopsy can cause rapid apoptosis and/or tissue necrosis, resulting in non‐diagnostic biopsies. Dural‐based lesions, although more easily accessible, can pose diagnostic difficulty due to crush artefact caused by dense fibrous tissue. As a diagnostic gold standard, detailed evaluation of the tissue biopsy using a wide range of immunohistochemical markers (supplemented where feasible by molecular diagnostics, fluorescent in situ hybridisation [FISH] studies and cytogenetics) and review by experienced haematopathologists is essential.

**FIGURE 1:**
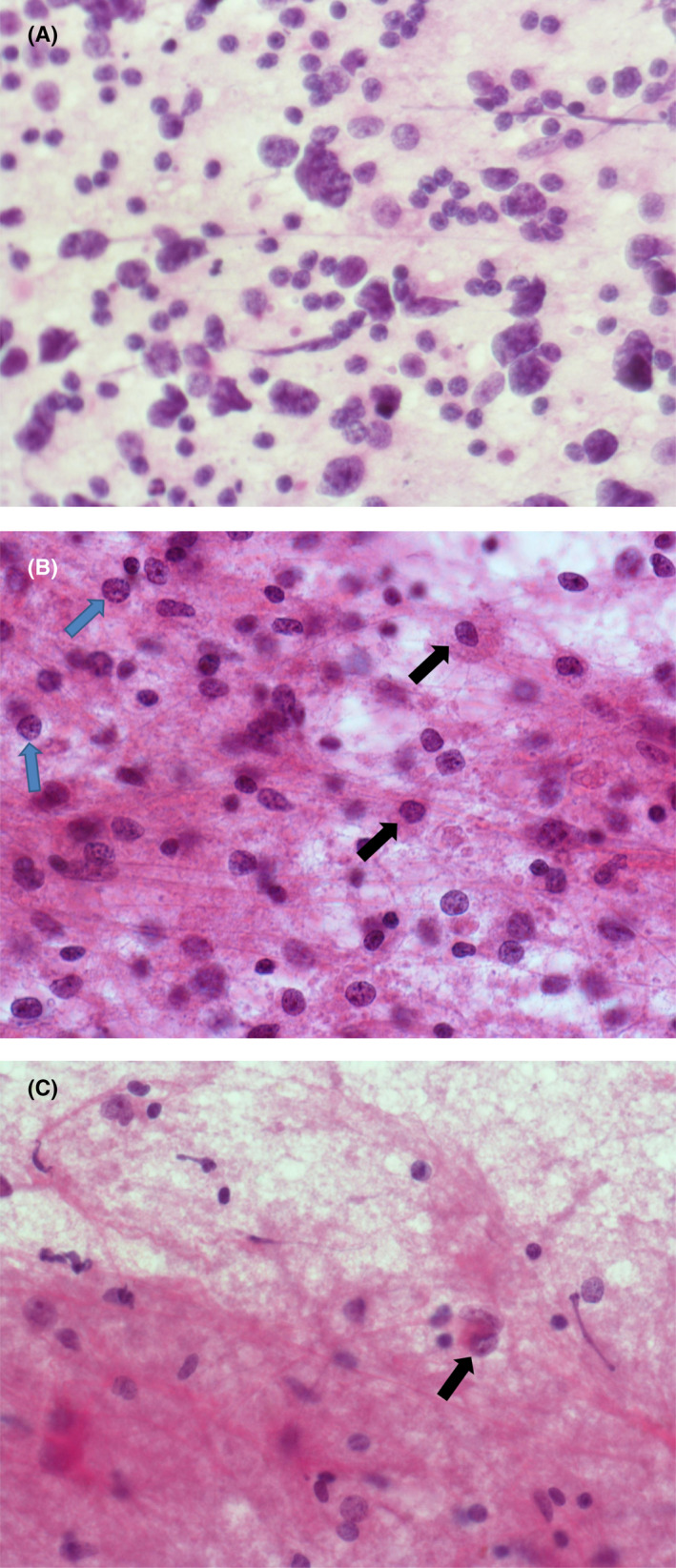
(A) A cellular touch preparation from an intraoperative procedure showing numerous atypical cells. Many cells have lost their cytoplasm, so called ‘naked nuclei’ (×50 oil immersion). (B) Touch preparation demonstrating glial proliferation masking neoplastic cells, which may be misinterpreted as a glial tumour. Glial cells are marked with black arrows and neoplastic cells with blue arrows (×50 oil immersion). (C) Pauci‐cellular touch preparation; the scant cells present are easy to identify as neoplastic due to their very abnormal chromatin pattern (see black arrow). The pink fibrillary background is normal astroglial tissue within the brain (×50 oil immersion)

Intraoperative smear for any primary CNS space occupying lesion is a common initial procedure undertaken in many neurosurgical units. The cytological features of lymphoma are similar to those seen from other anatomical sites with a population of non‐cohesive cells and presence of numerous ‘naked’ nuclei as a result of loss of delicate cytoplasm from these lymphoid cells (Figure 1A). Sometimes, the presence of numerous reactive astrocytes/reactive glial cells in the background can lead to misdiagnosis of a glial tumour (Figure 1B).^13^ The paucity of tumour cells within a non‐neoplastic background may be easily missed on cytology preparations (Figure 1C) resulting in diagnostic delays with potential clinical sequelae.

## SUBTYPES OF RARE CNS LYMPHOMA

**FIGURE 2:**
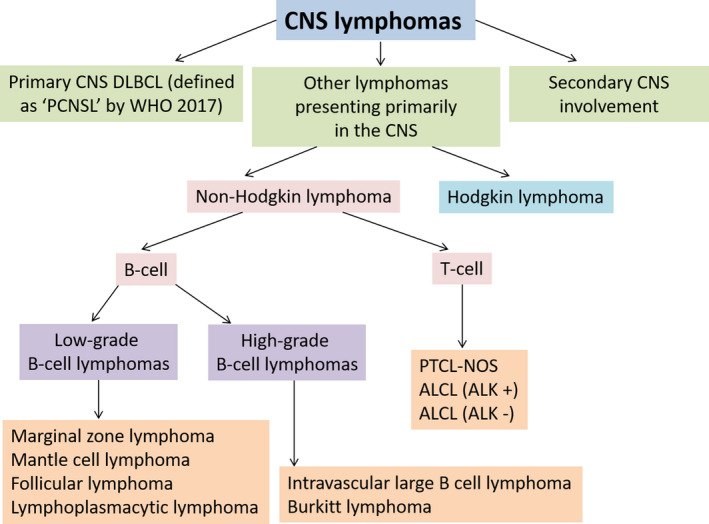
Histopathological classification of lymphomas presenting with central nervous system involvement. ALCL, anaplastic large cell lymphoma, ALK, anaplastic lymphoma kinase; DLBCL, diffuse large B‐cell lymphoma; PCNSL, primary central nervous system lymphoma; PTCL‐NOS, peripheral T‐cell lymphoma not otherwise specified; WHO 2017, World Health Organisation 2017 definition

Histopathological classification of rarer CNS lymphomas essentially follows the same diagnostic algorithms as their systemic counterparts. Figure 2 illustrates the essential histopathological delineation, focussing on the most frequent subtypes within this heterogeneous group of non‐DLBCL lymphomas that can present with dominant CNS disease.

### Hodgkin lymphoma

Primary and secondary CNS involvement in Hodgkin lymphoma (CNS‐HL) occurs in an estimated *≤*0.02%–0.5% of HL cases.^14^, ^15^, ^16^ Secondary disease is more common than primary involvement.^17^ The typical age at presentation of CNS‐HL appears to be 40–60 years. A large international multicentre case series, describing details of primary and secondary CNS‐HL, reported a median age of onset of 45 years.^17^ However, other studies report older median ages for patients with only primary CNS‐HL.^18^ For those with secondary involvement, CNS‐HL lesions presented at a median time of 11.7 months following initial diagnosis.^17^

CNS‐HL commonly presents as parenchymal disease, although dural‐based lesions have been reported.^19^, ^20^ Most intracranial lesions are supratentorial but infratentorial (including cerebellar) lesions have also been described.^19^ Similar to systemic HL, CNS‐HL may be associated with immunosuppressive states (including human immunodeficiency virus infection) and/or Epstein–Barr virus infection.^21^, ^22^, ^23^ Due to limited data, it is difficult to conclude whether either of these virus co‐factors are associated with an increased risk of CNS‐HL.^17^

The histopathological diagnosis of CNS‐HL is identical to that of systemic HL and reliant on identification of Hodgkin and Reed–Sternberg cells (expressing CD15, CD30, and multiple myeloma oncogene 1 [MUM1] with variable expression of B‐cell antigens) on a typical non‐neoplastic background comprising small lymphocytes, plasma cells, macrophages, and eosinophils.^18^, ^24^ In CNS‐HL, nodular sclerosing and mixed cellularity subtypes tend to predominate.^17^, ^19^, ^25^

For patients with primary CNS‐HL, surgical resection followed by radiotherapy (RT) is commonly reported to result in favourable outcomes in case reports. A case series of 16 CNS‐HL patients reported that 12/16 (75%) were treated with surgical resection followed by RT; most received WBRT, whilst the remainder received focal RT to specific disease sites.^18^ There was no evidence of residual or recurrent CNS‐HL in 90% of these patients with an average follow up time of 28 months. Indeed, one patient had no evidence of disease recurrence 10 years after local RT.^18^ Three of the 16 patients received chemotherapy (cyclophosphamide, vincristine, procarbazine and prednisone or MTX) in addition to surgery and RT with no evidence of disease after a median follow up of 14 months. Favourable outcomes have also been described in more recent case reports of patients treated with similar strategies.^26^, ^27^

Reported treatment approaches for secondary CNS‐HL are similar to those for primary CNS‐HL, with a greater tendency to use chemotherapy (with or without RT). Immune checkpoint inhibitors, particularly programmed cell death protein 1 (PD‐1) and programmed death‐ligand 1 (PD‐L1) inhibitors, have an established role in the clinical management of relapsed and refractory systemic HL, raising the possibility of their utility in CNS‐HL.^28^ Isolated case reports suggest that conventional HL chemotherapy protocols (including COPP/ABV [cyclophosphamide, vincristine, prednisone, procarbazine, doxorubicin, bleomycin, and vinblastine], ABVD [doxorubicin, bleomycin, vinblastine, and dacarbazine] and ICE [ifosfamide, carboplatin, and etoposide]) in addition to intrathecal MTX (IT‐MTX) can confer complete remission for many patients.^17^, ^29^, ^30^ However, further follow up in larger series is required to provide confidence in the rate and durability of the reported outcomes.

### Non‐Hodgkin lymphoma

#### Low‐grade B‐cell lymphomas

##### Marginal zone lymphoma

Amongst the different subtypes of low‐grade lymphoma presenting with CNS disease, marginal zone lymphoma (CNS‐MZL) is considered to be the commonest.^31^ A summary of recently published retrospective analyses of CNS‐MZL cases is presented in Table 1, most of which focus on dural involvement by MZL.

**TABLE 1:** Summary of recent retrospective analyses of central nervous system marginal zone lymphoma (CNS‐MZL) cases

Study	Primary/secondary CNS‐MZL	Number of cases	Average age at diagnosis, years, (range)	Sex distribution	Most common disease site	Treatments and survival outcomes
Sunderland et al. (2020)^34^	Primary and secondary	Primary (*n* = 13), secondary (*n* = 13)	59 (26–78) [median]	Primary: 69% female, 31% male Secondary: 54% female, 46% male	Dural	Most primary CNS‐MZL treated with RT ± CTx ± surgery (62%). Most secondary disease treated with CTx ± surgery (54%) 2‐year OS rates were 100% (primary CNS‐MZL) and 58% (secondary CNS‐MZL)
de la Fuente et al. (2017)^44^	Primary	Primary (*n* = 26)	50 (30–77) [median]	74% female, 26% male	Dural^a^	54% treated with RT + surgery. 23% treated with RT alone. 3‐year PFS was 89% and all patients alive at last follow up
Bayraktar et al. (2010)^54^	Primary and secondary	Primary (*n* = 6), secondary (*n* = 4)	Primary: 47 (29–71) [median] Secondary: no average (52–78)	Primary: 50% female, 50% male Secondary: not known	Dural	Primary CNZ‐MZL treated with RT alone (33%), CTx alone (33%), or surgery + CTx + RT (17%) Remainder not known Secondary CNZ‐MZL treated with RT alone (25%), CTx alone (25%), or surgery + CTx + RT (50%) All patients not lost to follow‐up/currently undergoing treatment achieved complete remission after treatment
Iwamoto et al. (2006)^50^, ^51^	Primary	Primary (*n* = 7)	49 (33–64) [median]	86% female, 14% male	Dural^a^	29% treated with RT alone, 29% with surgery + RT, and 43% with CTx + RT All patients achieved complete remission after treatment. Four patients relapsed/progressed within a year of treatment
Tu et al. (2005)^41^	Primary	Primary (*n* = 15)	55 (29–70) [mean]	80% female, 20% male	Dural	13% received CTx alone, 40% received RT alone, 7% received CTx + RT. Missing data for the remainder of patients All patients achieved clinical remission post treatment

Abbreviations: CTx, chemotherapy; OS, overall survival; PFS, progression‐free survival; RT, radiotherapy.

^a^ Only dural lymphomas were selected for consideration in these studies.

By contrast to CNS‐DLBCL, which tends to present more commonly in males, a recent systematic review of the literature found that 77% of reported cases of CNS‐MZL affect female patients.^31^, ^32^, ^33^ The estimated median age at diagnosis was 55 years (range: 18–78), considerably younger compared to patients with CNS‐DLBCL.^31^ More recent retrospective analyses have suggested that median age at diagnosis may be younger in patients with primary CNS‐MZL (51 years) compared to those with secondary CNS disease (62 years).^34^

Systemic MZL, particularly extra‐nodal subtypes outside the CNS, are often associated with chronic infectious or inflammatory processes (e.g. *Helicobacter pylori* infection in the stomach, Hashimoto's thyroiditis, and Sjögren's syndrome). Case reports have described instances of patients with CNS‐MZL who have these associated conditions,^35^ although a causal link has not been established. One hypothesis is that CNS‐MZL may be a direct consequence of aseptic meningitis (caused by enteroviruses, herpes simplex virus‐2, autoimmune phenomena, amongst others) or trauma, which induces lymphocytic recruitment to the leptomeninges.^36^ However, evidence to substantiate this hypothesis has not been forthcoming.

Extra‐nodal MZLs of the CNS predominantly present as dural‐based lesions, although parenchymal masses are also recognised, particularly in the context of secondary disease.^31^, ^34^ Tumour margins are often well defined on magnetic resonance imaging (MRI). Dural masses commonly display variable signal intensity on diffusion‐weighted MR sequences in relation to adjacent white matter.^37^

Given their anatomical predilection for the dura, CNS‐MZL are commonly mistaken for meningiomas on initial diagnostic imaging.^38^, ^39^, ^40^ However, the differential diagnosis for dural‐based lesions is wide, encompassing subdural haematomas, other tumours (e.g. dural metastases, glioma, leiomyosarcoma, plasmacytoma, schwannoma), and inflammatory lesions (e.g. pseudotumours, vasculitides, neurosarcoidosis).^31^, ^41^ As such, biopsy is required for a conclusive diagnosis particularly if there is no history of systemic MZL. It should be noted that composite meningiomas and CNS‐MZL have been described, with evidence of MZL invasion of the meningioma,^42^ further underscoring the importance of histopathological diagnosis of dural‐based lesions.

**FIGURE 3:**
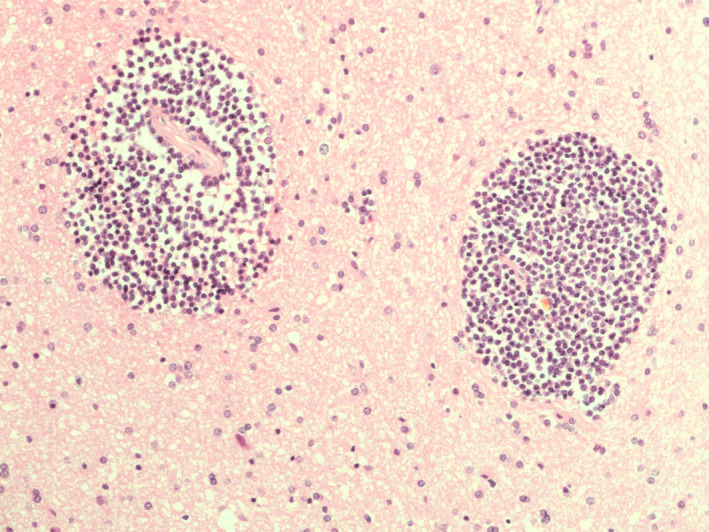
Perivascular lymphoid infiltrate comprising small lymphoid cells as seen on a low power field (×20 magnification)

Histopathological diagnosis of low‐grade B‐cell CNS lymphomas is often more challenging than for DLBCL, requiring more extensive immunohistochemistry panels for accurate sub‐classification. This is reflected in the largest series reported to date where a large majority (62.5%) were unclassifiable due to lack of adequate tissue.^43^ Perivascular patterns of infiltration, well described in primary CNS‐DLBCL, are also observed in low‐grade disease (Figure 3).

Similar to their systemic counterparts, marginal zone B cells express pan B‐cell markers (CD20, CD79A, CD19, CD22 and paired box 5 [PAX5]) and are typically CD5 and CD10 negative.^44^ Expression of immune receptor translocation‐associated protein 1 (IRTA1) has been reported as a useful marker for differentiating MZL from CD10‐negative follicular lymphoma.^45^ MZL outside the CNS frequently features IGH locus gene translocations, resulting in chimeric genes that can be identified within neoplastic B cells via FISH.^46^, ^47^, ^48^ By comparison, one of the largest studies on CNS‐MZL reported trisomy 3 as a common genetic abnormality in primary CNS‐MZL rather than IGH translocation.^41^

Active observation only (often referred to as ‘watch and wait’) may be appropriate for a number of years in asymptomatic patients with primary dural CNS‐MZL.^40^ Symptomatic primary disease has often been managed using surgical resection with or without RT.^44^, ^49^, ^50^ Complete surgical resection may be feasible for single discrete lesions, particularly if dural‐based, mindful of surgical risks and recognising that RT (focal or WBRT) can be highly effective. Moderate total radiation doses (e.g. 20–24 Gy) can achieve good responses whilst minimising neurotoxicity.^51^ Reported survival outcomes for patients treated with resection and/or RT are encouraging. In one study of 26 patients with primary dural CNS‐MZL (of whom 54% were treated with both RT and surgery, and 23% with RT alone), 3‐year progression‐free survival (PFS) was 89% (95% confidence interval [CI] 0.64–0.97) and all patients were alive at last follow‐up.^44^ These data are supported by a more recent report that described 2‐year overall survival (OS) rates of 100%, although this included a small number of patients treated with chemotherapy (BR [bendamustine and rituximab], R‐CHOP [rituximab, cyclophosphamide, doxorubicin, vincristine, and prednisolone] or rituximab +/− MTX) in addition to those who received surgery and RT.^34^

Treatment approaches for parenchymal disease, CNS‐MZL at relapse, or those with concurrent systemic disease are less well characterised in the literature. By contrast to published patterns of care for primary dural CNS‐MZL, treatment approaches for secondary CNS‐MZL have commonly included pharmacological therapies with or without surgical intervention.^34^, ^44^ A recent case series of secondary CNS‐MZL reported data from seven cases treated heterogeneously including RT, IT therapy (MTX, rituximab), combination systemic chemotherapy (CHOP, CVP [cyclophosphamide, vincristine, and prednisolone]), systemic high‐dose MTX, and intravenous rituximab in various combinations and schedules.^52^ Five out of seven patients achieved complete responses to treatment for ≥10 months.^52^ Earlier published studies on secondary CNS‐MZL describe a largely indolent clinical course with many patients free from disease progression for several years after treatment.^52^, ^53^, ^54^ However, a more recent observational study has highlighted inferior 2‐year OS rates for secondary CNS‐MZL (58%) compared to primary CNS disease (100%), underscoring the need for more data on this rare lymphoma subtype.^34^

##### Mantle cell lymphoma

Mantle cell lymphoma (MCL) is a distinct clinicopathological entity with heterogeneous clinical behaviour. A proportion of patients undergo an aggressive disease course, whilst the vast majority experience disease recurrence after a period of remission following therapy.^55^ Most cases of CNS disease involving MCL occur in the context of relapsed disease with an estimated reported frequency at relapse of 4.1%–7.8%.^56^, ^57^, ^58^ Typically, CNS‐MCL occurs as a relatively late event following initial therapy. Estimated median times from first diagnosis to CNS‐MCL range from 12 to 61 months.^56^, ^59^, ^60^

Both parenchymal and leptomeningeal CNS‐MCL have been reported.^56^, ^59^, ^61^ Risk factors for developing CNS relapse include blastoid histology,^56^, ^58^ raised serum LDH and high proliferative index.^56^, ^62^ Although CNS prophylaxis is not routinely recommended, some experts suggest this may be considered for patients with risk factors for CNS relapse.^55^, ^57^, ^62^ However it should be recognised that younger patients typically receive high‐dose cytarabine (HD‐AraC), integral to many first‐line treatment protocols.^55^, ^63^

**FIGURE 4:**
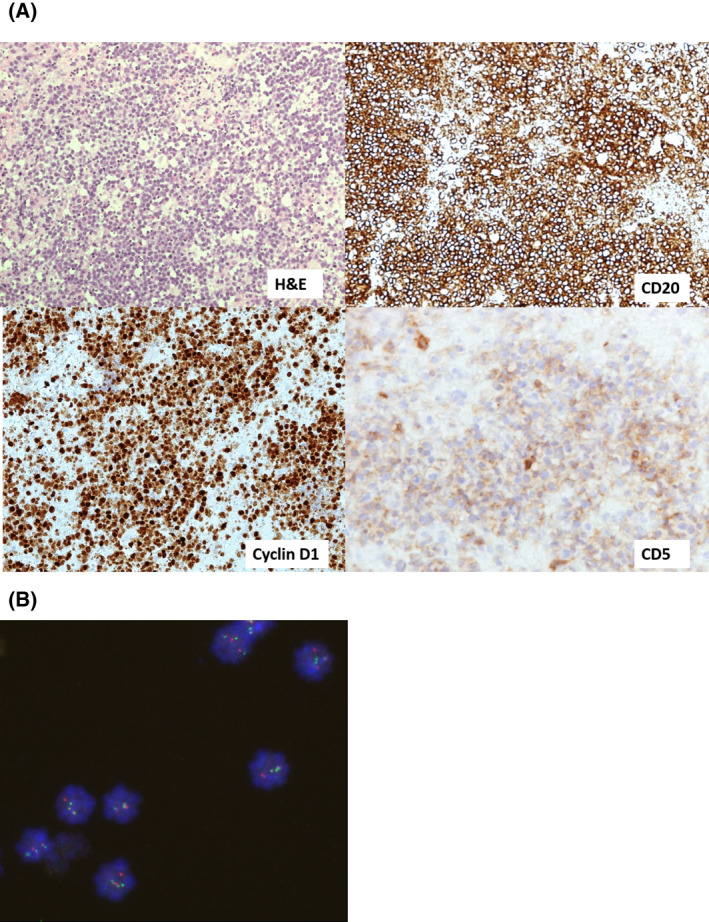
(A) A case of isolated central nervous system mantle cell lymphoma (CNS‐MCL) showing blastoid morphology. Cells are positive for CD20, CD5 and Cyclin D1 on immunohistochemistry (×20 magnification). H&E, haematoxylin and eosin staining. (B) Fluorescent in situ hybridisation image showing red‐green fusion signal consistent with immunoglobulin heavy locus‐Cyclin D1 (*IGH‐CCND1*) translocation. A normal signal is shown by two red and two green dots; red‐green fusion with one red dot (chromosome 11) and one green dot (chromosome 14) confirms t(11;14) rearrangement

CNS‐MCL, similar to its systemic counterpart, consists of a CD5^+^ B‐cell population expressing Cyclin D1 (encoded by the *CCND1* gene) as demonstrated in Figure 4A.^64^ FISH analysis for *t* (11;14)(q13;q32) involving the *IGH* and *CCND1* genes is recommended, particularly when morphology or immunophenotype is atypical (Figure 4B).^65^

Treatment approaches and clinical outcomes described in the literature have evolved over time. Earlier studies reported very poor outcomes, with a median survival in the order of 3–4 months from diagnosis of CNS relapse.^56^, ^57^ An international retrospective study of 57 patients with CNS‐MCL from fourteen different centres reported that 72% of patients received chemotherapy alone (HD‐MTX and/or AraC alone, or as part of R hyper‐CVAD [cyclophosphamide, vincristine, doxorubicin, dexamethasone, MTX, and AraC] or maxi‐CHOP).^57^ The remainder received chemotherapy and RT, RT alone or palliative care. IT administration of chemotherapy also featured commonly within reported regimens. A proportion of patients underwent ASCT as consolidation using carmustine (BCNU)‐etoposide‐AraC‐melphalan (BEAM) or bulsulfan‐melphalan conditioning.^57^ In the context of CNS‐MCL, superior OS rates (hazard ratio [HR] for death 0.42, 95% CI 0.19–0.91, *p* = 0.03) and sustained remission for >12 months were observed in patients consolidated with ASCT compared to non‐transplanted patients, although transplanted patients were generally younger with a superior baseline performance status.^57^ Based on these limited data, a reasonable treatment option for suitably fit patients with CNS‐MCL may include consolidation with high‐dose CNS‐penetrant chemotherapy (e.g. thiotepa, BCNU, or busulfan) and ASCT, extrapolating from experience with CNS‐DLBCL. However, it must be acknowledged that ASCT (typically with BEAM conditioning) is an established consolidative strategy in first response for many patients aged <65 years with systemic MCL.^55^

More recently the Bruton's tyrosine kinase inhibitor (BTKi), ibrutinib, approved for the treatment of relapsed/refractory MCL,^66^ has been explored as a possible treatment option for CNS‐MCL given its ability to cross the blood–brain barrier.^61^ A recent summary of five cases treated with ibrutinib (560 mg once daily) alone, or in combination with other chemotherapeutic agents and/or steroids, described objective clinical responses in all patients within 2 weeks. However, the durability of response was short (median duration of response: 4 months).^67^ Other data indicate more encouraging outcomes with sustained complete response rates for up to 2 years.^61^, ^68^ Although these data are preliminary, it appears that ibrutinib may be an effective and well‐tolerated treatment alternative for CNS‐MCL, acknowledging the lack of durability in most cases.^67^

##### Follicular lymphoma

Descriptions of primary and secondary CNS follicular lymphomas (CNS‐FL) are scarce within the literature. In common with the other rare CNS lymphomas described in this review, case reports dominate the literature on CNS‐FL.

Both primary and secondary CNS‐FL^69^, ^70^, ^71^, ^72^, ^73^ have been described, although transformation into high‐grade B‐cell non‐Hodgkin lymphoma (B‐NHL) must always be considered in the context of secondary CNS disease.^70^, ^71^ Similar to CNS‐MZL, most cases of low‐grade CNS‐FL describe a dural pattern of involvement, such that they may be mistaken for meningiomas.^37^, ^74^, ^75^ However, FLs represent a low proportion of dural lymphomas overall; these are predominantly CNS‐MZL.^76^, ^77^

Histopathologically, CNS‐FL can manifest as a nodular or diffuse pattern and show a mixed population of centrocytes and centroblasts that typically co‐express CD10 and BCL6. BCL2 overexpression, the hallmark of FL, is seen commonly in low‐grade disease or can at times be negative.^78^

Treatment approaches adopted for CNS‐FL have typically included surgical resection followed by chemotherapy and/or RT.^75^ By corollary to early‐stage low‐grade systemic FL, often treated with RT alone, isolated dural tumours have been treated with RT as a single modality, associated with good survival outcomes.^69^, ^79^ Transformation of FL into high‐grade B‐NHL of the CNS is usually treated using existing CNS‐DLBCL protocols. For patients with concurrent CNS and systemic involvement by low‐grade FL, intravenous (with/without IT) chemotherapy has been associated with favourable clinical outcomes. Regimens reported in the literature include various combinations of HD‐MTX, IT‐MTX, AraC, rituximab, CHOP, and CVP with or without RT.^70^, ^72^, ^75^ Anthracycline‐containing regimens alternating with MTX have been employed to target both systemic and CNS disease respectively.^72^, ^80^ However, it should be recognised that, given the typically low proliferation rate of low‐grade FL cells, anti‐metabolite chemotherapy agents such as MTX and AraC may be less effective than for CNS‐DLBCL. Bendamustine, commonly used for systemic FL, is a potentially effective option due to its ability to cross the blood–brain barrier, although this is not frequently described as a therapy in the published literature for CNS‐FL.^81^ Similarly, combination lenalidomide (a CNS‐penetrating agent) and rituximab, already used in relapsed and refractory systemic FL, may be a reasonable option in secondary CNS‐FL.^82^, ^83^ Some clinicians report using maintenance rituximab due to superior PFS rates observed in systemic FL.^80^, ^84^ Of the few cases of concurrent systemic and CNS‐FL reported in the literature, most patients achieved clinical remission.^70^, ^72^, ^75^, ^80^

Dural lymphomas in particular have a good prognosis with complete surgical resection.^79^ A database analysis of >4000 patients with primary CNS lymphoma diagnosed between 1998 and 2014 suggest that the 5‐year OS rate for patients with primary CNS‐FL is significantly higher compared to those with primary CNS‐DLBCL (66% [95% CI 54%–76%] vs. 30% [95% CI 28%–32%]). This was confirmed in multivariate analysis, adjusted for age and treatment type (HR 0.32, 95% CI 0.23–0.46, *p* < 0.001 – compared to DLBCL),^85^ although survival outcomes for CNS‐DLBCL have improved in recent years.^5^, ^86^

##### Lymphoplasmacytic lymphoma (Waldenström macroglobulinaemia)

Lymphoplasmacytic lymphoma (LPL) or Waldenström macroglobulinaemia (WM) is a low‐grade lymphoproliferative disorder, resulting in the production of a monoclonal immunoglobulin M (IgM) paraprotein by lymphoplasmacytoid cells that infiltrate the bone marrow.^87^ Although neurological signs and symptoms may occur in the context of LPL, they are not always the direct result of neoplastic infiltration into the CNS. The spectrum of neurological features in LPL/WM includes:

1. IgM neuropathy: a demyelinating neuropathy caused by monoclonal IgM activity against myelin associated glycoprotein, resulting in proprioceptive and sensory dysfunction.^88^
2. Hyperviscosity syndrome: high levels of IgM paraprotein in the plasma resulting in increased viscosity, manifesting as headaches, retinopathy, seizures, and altered conscious level.^89^
3. Transformation to CNS high‐grade B‐NHL: typically a subacute presentation akin to PCNSL and treated with CNS‐DLBCL protocols.
4. Bing–Neel syndrome (BNS): neoplastic lymphoplasmacytic cells directly invade the blood–brain barrier, often with leptomeningeal involvement, causing a spectrum of central neurological phenomena.
5. Primary CNS‐LPL (PCNS‐LPL): characterised by lymphoplasmacytoid cells within the CNS without evidence of LPL in the bone marrow.

IgM neuropathy, hyperviscosity syndrome, and transformation to high‐grade lymphoma are outside the scope of this review. Thus, we have focussed on BNS and PCNS‐LPL.

##### Bing–Neel syndrome

Bing–Neel syndrome may be the first presentation of LPL or, more commonly, appear later in the disease course. Estimates of median time from LPL diagnosis to the development of BNS are around 3–4 years.^90^, ^91^

Contrast‐enhanced MRI of the neuroaxis commonly reveals leptomeningeal enhancement in ~80% of cases of BNS but is not pathognomonic.^92^ Mass lesions within the brain parenchyma are less common, but are recognised in the context of BNS.^93^, ^94^ Particularly where mass lesions are present, it is important to exclude histopathological transformation to an aggressive B‐cell lymphoma.

**FIGURE 5:**
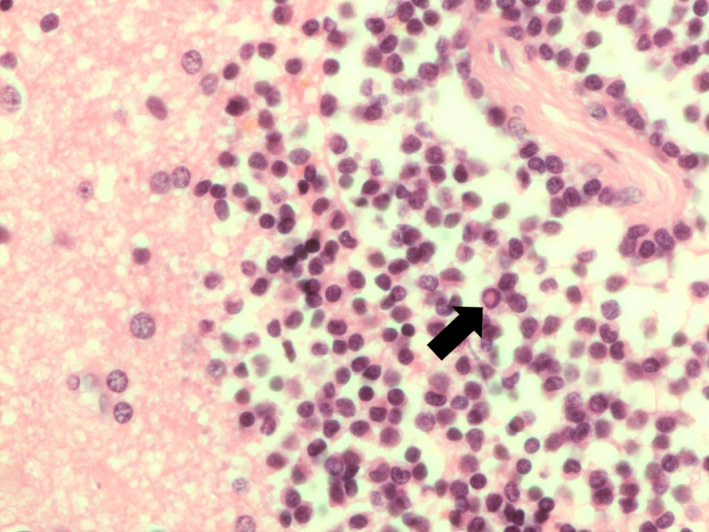
Monomorphic population of small‐to‐medium sized cells, some showing lymphoplasmacytic morphology. Occasional cells show intranuclear immunoglobulin inclusions (see arrow): ‘Dutcher bodies’ (×40 magnification)

Diagnosis of BNS requires the demonstration of clonal lymphoplasmacytoid cells within CSF and/or tissue biopsy of a mass within the CNS demonstrating features of LPL.^93^ Confirmation of LPL infiltration in the bone marrow is also helpful in securing the diagnosis.^92^ Microscopically, lymphoplasmacytoid cells typically display characteristic intranuclear Ig inclusions (Dutcher bodies) (Figure 5).^89^ They express pan‐B‐cell antigens (CD19, CD20, CD22, CD79a and PAX5), whilst being negative for CD5 and CD10. The plasma cells in LPL are CD138 positive as well as CD19, CD45 and PAX5 positive, unlike in plasma cell myeloma where the cells do not express CD45 or PAX5.^89^ Elevated IgM within the CSF and serum may also be observed in addition to a monoclonal IgM paraprotein on serum electrophoresis.^92^ Molecular analysis of cells within the CSF often reveals *IGH* gene rearrangements and *MYD88* mutations (L256P). Importantly, this must be correlated with clinical and radiological features as *MYD88* mutations are common in patients with primary CNS‐DLBCL and are also described in MCL and chronic lymphocytic leukaemia.^91^, ^92^, ^95^

Similar to other low‐grade B‐NHL subtypes, asymptomatic BNS may not require treatment and close monitoring is considered an acceptable management approach.^92^ Historically, conventional chemotherapy, often empirically adopted from PCNSL protocols, has been employed for symptomatic BNS, e.g. HD‐AraC and HD‐MTX. However, the toxicity of these anti‐metabolite chemotherapy agents may not be warranted, particularly given the lack of strong rationale for the treatment of indolent B‐NHL with low proliferative rates. Purine‐analogue and related agents (e.g. fludarabine, bendamustine), with established efficacy for systemic LPL, have also been employed for BNS often in combination with rituximab, but these regimens can be potently immunosuppressive and are usually reserved for relapsed disease in fitter patients.^92^, ^96^, ^97^ One study estimated an overall response rate of 70% after treatment with chemoimmunotherapy with an OS of 71% at 5 years,^90^ although treatment approaches were very heterogeneous and no clear conclusions could be made regarding the relative efficacy of the different management strategies. Some clinicians propose that ASCT may prolong remission after first‐line treatment for BNS. A recent review found that of 14 patients who underwent ASCT (with either BEAM or thiotepa‐based conditioning regimens), 13 remained in remission after a median follow up of 35 months.^98^

More recently, ibrutinib, licensed for the treatment of systemic LPL, has emerged as a potential treatment option for BNS, owing to its efficacy, oral administration and ability to penetrate the blood–brain barrier.^99^, ^100^, ^101^, ^102^ In one study, 2‐year event‐free survival (measured from the time of treatment initiation to disease progression, treatment toxicity or death from any cause) was 80%.^92^ Although second‐generation BTKi have shown promise in treating systemic LPL,^103^ we found no reported data on these agents in the context of BNS.

##### Primary CNS‐LPL

Distinct from BNS is PCNS‐LPL, where lymphoplasmacytoid cells are present in the CNS but not elsewhere systemically, including the bone marrow.^104^ Combined morphological and immunophenotypic assessments have improved the ability to exclude lymphoplasmacytic infiltration into the bone marrow.^105^ More recently, this has been enhanced with the development of specific polymerase chain reaction assays used to detect *MYD88* mutations in bone marrow cell populations, with high sensitivity.^106^ Descriptions of PCNS‐LPL within the literature are rare when compared to BNS and, as such, there is no consensus on treatment strategies. Several case reports describe successful treatment with WBRT alone^104^, ^107^ but the frequent leptomeningeal involvement with LPL cells may not be sufficiently addressed by this strategy. Another report described successful treatment with surgical resection, chemotherapy and RT, inducing complete remission for 4 years.^108^

### High‐grade B‐cell lymphomas

#### Intravascular large B‐cell lymphoma

Intravascular large B‐cell lymphoma (IVL) is a rare high‐grade B‐cell malignancy characterised by an almost exclusive growth of malignant cells within peripheral blood vessels.^109^ Estimated incidence is <1 case per million per annum.^110^ Up to 61% of IVL cases with CNS involvement are diagnosed post‐mortem, implying late presentation and/or diagnosis with rapidly progressive neurological deterioration.^111^

CNS involvement has been described in 30%–40% of patients at diagnosis (although this may be an underestimate given the potential for subclinical disease), whilst a further 25% develop CNS disease during follow‐up.^112^ Neurological manifestations are usually accompanied by systemic phenomena, of which skin infiltration is the most frequent.^113^

There are no typical diagnostic radiological findings. MRI is frequently normal, but may show diffuse multifocal white matter involvement, infarct‐like lesions, meningeal and focal nodular parenchymal enhancement.^114^, ^115^, ^116^, ^117^ As such, IVL with CNS involvement should be included in the differential of rapidly progressive neurological symptoms with ischaemic MRI changes in the absence of cardiovascular risk factors.^118^ Positron emission tomography has been described as a potentially useful diagnostic tool,^119^ particularly the ^11^C‐methionine radionuclide, which seems to be able to identify IVL CNS lesions when MRI appearances resemble vasculitis.^120^

**FIGURE 6:**
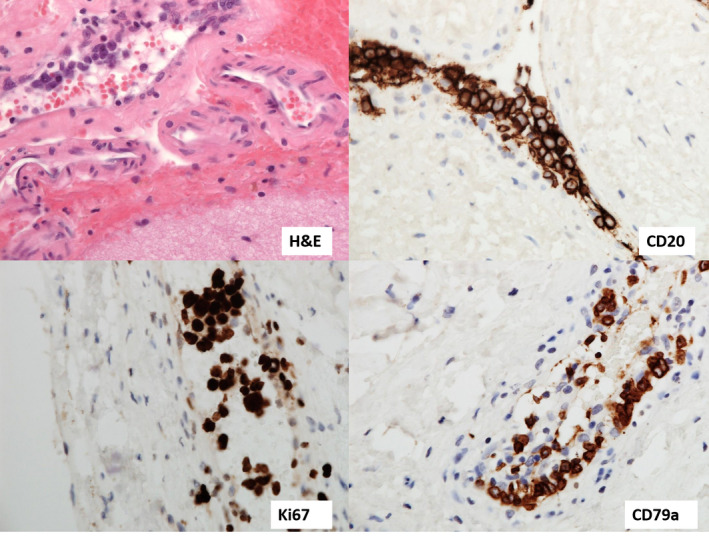
Brain biopsy tissue demonstrating the intravascular location of large atypical cells is demonstrated in the top left panel (red blood cells within the blood vessel provide a good size comparison). Cells are positive for CD20 and multiple myeloma oncogene 1 (MUM1) and show high proliferative fraction on Ki67 staining (×40 magnification). H&E, haematoxylin and eosin staining

CSF examination offers little aid in diagnosis, as neoplastic cells are frequently absent.^121^, ^122^ Brain biopsy histopathology reveals arrays of intravascular CD20 cells with MUM1 expression in 75%–80% of cases (Figure 6).^113^ Recent reports have highlighted the potential use of *CD79B* Y196H and *MYD88* L256P mutations as diagnostic aids using plasma ctDNA (detected in 26% and 44% of IVL cases respectively).^123^

Given the disease biology and need for rapid clearance of tumour cells, systemic chemotherapy is often the preferred treatment modality. R‐CHOP is most frequently used, demonstrating good outcomes; within a cohort of 10 patients in which half had CNS disease, 3‐year OS rates were 81%.^124^, ^125^ A recent population‐based analysis described 5‐year OS rates of 46%.^126^ Systemic CNS‐directed therapy of isolated CNS‐IVL has been reported with the use of the MTX‐containing Bonn‐Protocol (MTX, ifosfamide, procarbazine, AraC, vincristine, dexamethasone, and rituximab),^127^ BCNU‐AraC‐MTX,^128^ HD‐MTX followed by RT, and the deAngelis protocol (MTX, vincristine, procarbazine, and rituximab).^129^ RT has been seldom used in isolated CNS‐IVL, with disappointing results.^121^, ^130^ Although the number of reported cases is too small to conclude that WBRT is ineffective, this is not considered a rational approach given the biology of IVL.

For patients without CNS disease at IVL diagnosis, CNS prophylaxis remains a key aspect of IVL management. Although data are limited; delivery of CNS prophylaxis is justified given the prevalence and risk of CNS disease in IVL.

#### Burkitt lymphoma

Burkitt lymphoma (BL) is a distinct clinicopathological B‐cell lymphoma entity with aggressive clinical behaviour, associated with a high risk of CNS involvement (CNS‐BL) ranging from 5% to 40%.^131^, ^132^, ^133^ Standard therapy for systemic BL involves blood–brain barrier penetrating systemic chemotherapy and IT chemotherapy e.g. R‐CODOX‐M/R‐IVAC (rituximab, cyclophosphamide, vincristine, doxorubicin, MTX, ifosfamide, etoposide, and AraC).^134^ Isolated CNS‐BL has been reported in <40 cases in the literature to date.^135^, ^136^ Gadolinium enhancing white matter lesions are frequently reported on MRI, although other unusual manifestations have been described, such as oculomotor nerve palsy in the absence of radiological or CSF manifestations.^137^, ^138^ Cases reported to date have predominantly employed HD‐MTX‐based treatment. However, the optimal combination regimen remains unknown. Disease‐free intervals of the reported cases range from 4 to 48 months.^139^, ^140^, ^141^, ^142^, ^143^, ^144^

### T‐cell lymphomas

Primary CNS T‐cell lymphomas (PCNS‐TCL) account for between 2% and 8.5% of all primary lymphomas within the CNS,^145^, ^146^, ^147^ with a reported higher incidence in Eastern countries.^148^ Estimates of average age at diagnosis range from 55.8 to 60 years.^149^, ^150^ Interestingly, a recent systematic review described a much lower median age at diagnosis for those with primary CNS anaplastic large cell lymphoma (PCNS‐ALCL), at 21 years (range 1–82 years).^151^

Mature peripheral TCLs comprise a spectrum of biologically and clinically heterogeneous malignancies. In two large retrospective studies of primary and secondary CNS‐TCL, the commonest histopathological subtype was peripheral TCL‐not otherwise specified (PTCL‐NOS) (83% and 54% respectively), with other subtypes also described; ALCL, angioimmunoblastic lymphoma, adult TCL/leukaemia, and extra‐nodal natural killer TCL.^147^, ^152^, ^153^ Due to extensive heterogeneity across the spectrum of secondary CNS‐TCLs, we have focussed our discussion on primary disease and PTCL‐NOS and ALCL subtypes, which constitute the majority of CNS‐TCL cases.^147^

**FIGURE 7:**
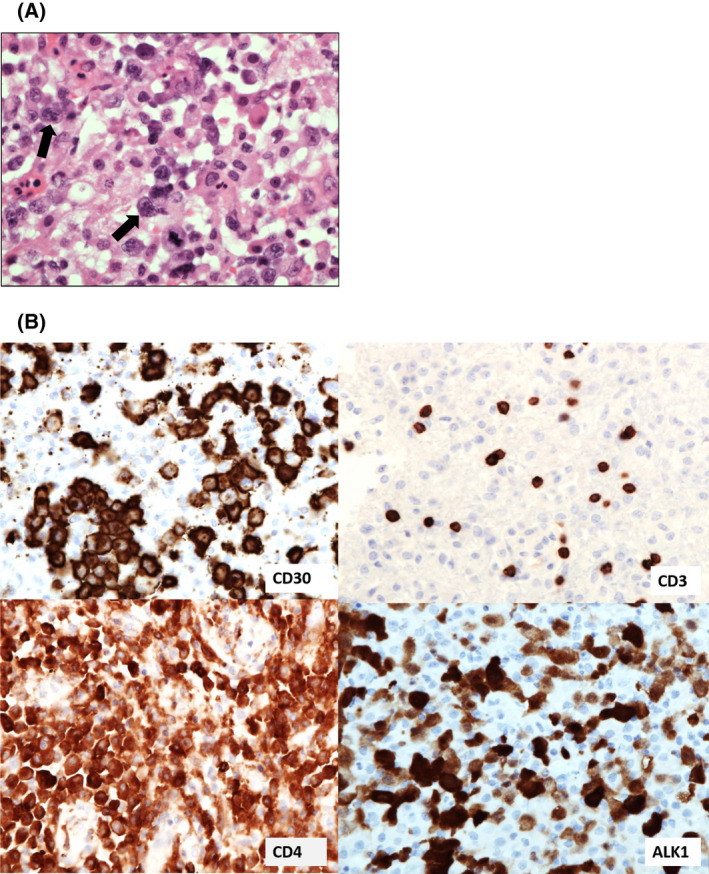
(A) Pleomorphic cells from a brain biopsy specimen demonstrating anaplastic large cell lymphoma with numerous bi‐lobed nuclei (see arrows) and prominent nucleoli (×40 magnification). (B) Cells show strong staining with CD30 (including Golgi staining) and anaplastic lymphoma kinase 1 (ALK1, nuclear and cytoplasmic staining). Large cells are negative for CD3 but stain with CD4 (×40 magnification)

There are no specific radiological features which clearly delineate CNS‐TCL from B‐NHL within the CNS. PCNS‐TCL may occur within the brain parenchyma or the leptomeninges. In particular, ALCL has a predilection for the meninges with one review suggesting 80% of reported PCNS‐ALCLs exhibit meningeal involvement.^151^ Morphologically, PCNS‐PTCL‐NOS can be difficult to distinguish from reactive T‐cell infiltrates and B‐cell lymphomas; these neoplasms may be CD4 or CD8 positive.^147^ By contrast, ALCL may manifest as large pleomorphic populations of cells with horse‐shoe shaped or multi‐lobated nuclei with vesicular chromatin and prominent eosinophilic nucleoli (Figure 7A). The diagnostic immunophenotype of ALCL is that of uniform cytoplasmic and Golgi staining with CD30 and loss of numerous T‐cell antigens.^147^ Similar to their systemic counterparts, PCNS‐ALCL may be anaplastic lymphoma kinase (ALK) positive or negative (Figure 7B).^151^

Owing to their rarity, no standard treatment protocol has been established for PCNS‐TCL. Similar to CNS‐DLBCL, most regimens described within the literature include systemic HD‐MTX and/or IT‐MTX.^148^, ^151^, ^154^, ^155^ Intravitreal MTX and local RT have been reported as treatment options for ocular involvement.^155^ For PCNS‐DLBCL, HDT‐ASCT has demonstrable efficacy as the preferred consolidation strategy. Accordingly, HDT‐ASCT represents a viable consolidative strategy in the context of PCNS‐TCL.^7^

Despite treatment, PCNS‐TCL confers a very poor prognosis. A large case series comprising a range of histopathological subtypes estimated patients with PCNS‐TCL have a short median OS of 8 months with a 3‐year OS of 32.8%.^150^ Specifically for patients with PCNS‐ALCL, a systematic review of reported cases found that ALK‐positive tumours were associated with superior 2‐year OS rates compared to ALK‐negative neoplasms (71% vs. 22%).^151^ However, this observation may be confounded by age; the ALK‐positive group was significantly younger than the ALK‐negative cohort (17.5 vs. 63 years).^151^

## A PRAGMATIC CLINICAL APPROACH TO RARE CNS LYMPHOMAS

For patients presenting with these rare primary and secondary CNS lymphoma entities, a diagnosis may not be immediately forthcoming. In Table 2, we summarise a non‐exhaustive list of possible signs and symptoms that may prompt clinicians to consider neuroimaging and/or CSF examination, particularly in patients with established systemic lymphoma. Additionally, we have summarised recommended investigations and staging procedures. Mindful of the fragile evidence‐base underpinning clinical management decisions for many of these rare lymphomas, we present rational treatment options informed by the available literature and our clinical experience of these entities.

**TABLE 2:** Summary of signs and symptoms, investigations (including staging), and possible management strategies for each lymphoma subtype

Clinical features (in the context of known systemic lymphoma)	Investigations	Possible management strategies^a^		
Focal upper motor neurone deficits Cranial nerve palsies Visual disturbance Cerebellar ataxia Radiculopathies Cognitive dysfunction (e.g. amnesia, dysphasia, dyspraxia) Persistent headache Symptoms of raised intracranial pressure Seizures	*Establish diagnosis of CNS disease*: MRI neuroaxis Tissue biopsy for histopathological examination Lumbar puncture – cytology, flow cytometry and molecular analysis *Exclude systemic disease*: Whole body PET‐CT (or contrast‐enhanced CT neck, chest, abdomen and pelvis) Bone marrow biopsy	Classical Hodgkin lymphoma		*Primary CNS‐HL*: Parenchymal infiltration: CNS‐penetrant chemotherapy protocols with established HL activity e.g. ICE Dural‐based: standard HL regimens e.g. ABVD. RT if not fit for chemotherapy or for residual disease^b^ *Secondary CNS‐HL*: CNS‐penetrant chemotherapy protocols with established HL activity e.g. ICE or standard HL regimens e.g. ABVD ± RT^b^
		B‐cell NHL	CNS‐MZL	*Primary CNS‐MZL*: Surveillance only (watch and wait) if incidental finding of an asymptomatic dural lesion. RT^b^ and/or surgical resection (particularly if symptomatic and dural‐based) Chemoimmunotherapy e.g. BR ± IT therapy (for parenchymal and/or leptomeningeal disease) *Secondary CNS‐MZL*: Chemoimmunotherapy e.g. BR ± IT therapy
			CNS‐MCL	HD AraC‐based protocol with thiotepa‐based ASCT consolidation BTKi Chemoimmunotherapy e.g. BR or RBAC ± IT therapy (if previous BEAM ASCT or ASCT‐ineligible)
			CNS‐FL	*Primary CNS‐FL*: Surveillance only (watch and wait) if asymptomatic or incidental finding RT^b^ or surgical resection (if symptomatic) *Secondary CNS‐FL*: Chemoimmunotherapy e.g. BR ± anti‐CD20 antibody maintenance Lenalidomide and rituximab
			BNS & PCNS‐LPL	Chemoimmunotherapy e.g. BR ± IT therapy Thiotepa‐based ASCT consolidation. BTKi RT (unifocal mass lesions)^b^
			IVL	Systemic chemotherapy, e.g. R‐CHOP and HD‐MTX or more intensive HD‐MTX containing regimens
			CNS‐BL	Established BL protocols with multiple CNS‐penetrant agents e.g. R‐CODOX‐M/R‐IVAC
		T‐cell NHL	PCNS‐TCL	HD‐MTX based CNS‐penetrating chemotherapy regimens (primary DLBCL‐CNS protocols, *without* rituximab) Thiotepa‐based ASCT consolidation

Abbreviations: ABVD, doxorubicin, bleomycin, vinblastine, and dacarbazine; AraC, cytarabine; ASCT, autologous stem cell transplantation; BEAM, carmustine, etoposide, cytarabine, and melphalan; BNS, Bing–Neel syndrome; BL, Burkitt lymphoma; BR, bendamustine and rituximab; BTKi, Bruton's tyrosine kinase inhibitor; CNS, central nervous system; CT, computed tomography; FL, follicular lymphoma; HD, high dose; HL, Hodgkin lymphoma; ICE, ifosfamide, carboplatin, and etoposide; IT, intrathecal; IVL, intravascular lymphoma; MCL, mantle cell lymphoma; MRI, magnetic resonance imaging; MTX, methotrexate; MZL, marginal zone lymphoma; NHL, non‐Hodgkin lymphoma; PCNS‐LPL, primary central nervous system lymphoplasmacytic lymphoma; PCNS‐TCL, primary central nervous system T‐cell lymphoma; PET, positron emission tomography; RBAC, rituximab, bendamustine, and cytarabine; R‐CHOP, rituximab, cyclophosphamide, doxorubicin, vincristine, and prednisolone; R‐CODOX‐M/RIVAC, rituximab, cyclophosphamide, vincristine, doxorubicin, methotrexate, ifosfamide, etoposide, and cytarabine; RT, radiotherapy; TCL, T‐cell lymphoma.

^a^ Available evidence supporting clinical management recommendations is weak. Possible management approaches listed here are based on published data and clinical experience of the authors. All treatments are off‐label for CNS disease. Clinical decision‐making should be made on a case‐by‐case basis, considering all patient‐ and disease‐related (including CNS compartment: dural vs. parenchymal vs. leptomeningeal) factors, supported by expert advice wherever possible.

^b^ RT dose and field should be discussed with an expert radiation oncologist. It is reasonable to adopt similar dose and fractionation schedules applied for the systemic lymphoma counterpart, but additional consideration should be given to whether the field is focal or whole‐brain, mindful of neurocognitive sequelae.

## CONCLUSIONS AND OUTLOOK

In this review, we have summarised the epidemiology, diagnostic features, reported management strategies, and anticipated survival outcomes across the rare CNS lymphomas. It is clear that there is substantial clinical, radiological and histopathological heterogeneity which, together with the rarity of these entities, presents a weak evidence‐base to support clinical decision‐making.

Approaches to clinical management of the low‐grade B‐cell CNS lymphomas, particularly CNS‐MZL and CNS‐MCL, are relatively better characterised due to larger numbers of published cases. However, conclusions regarding optimal management strategies for these and other rarer CNS lymphoma subtypes are limited by study design, small case numbers, potential for selection and publication bias, and heterogeneity of treatment approaches. As such, current management is often informed by existing protocols for their systemic lymphoma counterparts and/or empirically adopted from CNS‐DLBCL, together with rational application of chemotherapeutic agents known to penetrate the blood–brain barrier.

Looking ahead, emerging technologies such as ctDNA in plasma and CSF offer great promise as a paradigm‐shift in biological classification, as well as offering an opportunity for individualised, dynamic assessment of treatment response.^11^ Moreover, ctDNA should provide much greater sensitivity for the detection of concomitant subclinical disease in both systemic and CNS compartments. Importantly, ctDNA and sequencing technology, together with enhanced potential for discovery science on small and/or fixed tissue specimens, promises further opportunities to develop biologically‐rational therapies informed by a more precise understanding of subtype specific pathobiology.

Interventional studies specifically designed for distinct rare CNS lymphomas may be considered too challenging to deliver. Nevertheless, inclusion of such CNS cohorts within subtype‐specific systemic lymphoma protocols, with biologically rational therapies, is an important and achievable goal.

## CONFLICT OF INTEREST

Christopher Paul Fox has received speaker and consultancy fees from Roche, Adienne, Janssen, Astrazeneca and research funding from BeiGene.

## AUTHOR CONTRIBUTIONS

Christopher Paul Fox initiated and supervised the review. Furqaan Ahmed Kaji undertook the literature review. Furqaan Ahmed Kaji, Vishakha Sovani, Nicolás Martinez‐Calle and Christopher Paul Fox reviewed the literature summary, critically revised the manuscript and approved the final version.
